# Resistant starch supplementation effects on plasma indole 3-acetic acid and aryl hydrocarbon receptor mRNA expression in hemodialysis patients: Randomized, double blind and controlled clinical trial

**DOI:** 10.1590/2175-8239-JBN-2020-0003

**Published:** 2020-05-18

**Authors:** Renata Azevedo, Marta Esgalhado, Julie Ann Kemp, Bruna Regis, Ludmila FMF Cardozo, Lia S. Nakao, Jessyca Sousa de Brito, Denise Mafra

**Affiliations:** 1 Universidade Federal Fluminense, Programa de Pós-graduação em Ciências Cardiovasculares, Niterói, RJ, Brazil.; 2 Universidade Federal Fluminense, Programa de Pós-graduação em Ciências Médicas, Niterói, RJ, Brazil.; 3 Universidade Federal do Paraná, Programa de Pós-graduação em Ciências Médicas, Curitiba, PR, Brazil.

**Keywords:** Prebiotics, Aryl hydrocarbon receptor, Inflammation, Chronic kidney disease, hemodialysis, Prebióticos, Receptor aril hidrocarboneto, Inflamação, Doença renal crônica, hemodiálise

## Abstract

**Introduction:**

Gut microbiota imbalance is linked to high uremic toxins production such as indole-3-acetic acid (IAA) in chronic kidney disease patients. This toxin can activate the aryl hydrocarbon receptor (AhR), a ligand-activated transcription factor involved with inflammation. Strategies to restore gut microbiota balance can be associated with reduced production of IAA and its deleterious effects. This study aimed to evaluate prebiotic resistant starch (RS) supplementation effects on IAA plasma levels and AhR mRNA expression in CKD patients on hemodialysis (HD).

**Methods:**

This randomized, double-blind and placebo-controlled clinical trial evaluated forty-two stable HD patients allocated in RS (n=22) or placebo (n=20) groups. Patients received, alternately, cookies and sachets containing 16 g/day of RS (Hi-Maize 260^®^) or manioc flour for four weeks. Fasting pre-dialysis blood samples were collected and IAA plasma levels measured by high performance liquid chromatography. Peripheral blood mononuclear cells were isolated and processed for AhR and nuclear factor kappa B (NF-κB) mRNA expression analyzes by quantitative real-time PCR. Anthropometric and biochemical parameters, as well as food intake were also evaluated.

**Results:**

Thirty-one patients completed the study, 15 in the RS group and 16 in the placebo group. Although there was no significant alteration in IAA plasma levels, neither in AhR mRNA expression and NF-κB mRNA expression after RS supplementation, a positive correlation (r=0.48; p=0.03) was observed between IAA plasma levels and AhR expression at baseline.

**Conclusion:**

Even though prebiotic RS supplementation did not influence IAA levels or AhR expression, their positive association reinforces a possible interaction between them.

## RESUMO

**Introdução:**

O desequilíbrio da microbiota intestinal associa-se à alta produção de toxinas urêmicas tais como ácido indol-3-acético (AIA), em renais crônicos. Essa toxina ativa o receptor aril hidrocarboneto (AhR) - fator de transcrição ativado por ligante, na inflamação. Restaurar o equilíbrio da microbiota intestinal associa-se à produção reduzida de AIA e efeitos deletérios. Avaliamos os efeitos da suplementação de amido resistente prebiótico (AR) sobre AIA sérico e expressão de AhR mRNA em renais crônicos em HD.

**Métodos:**

Estudo clínico randomizado, duplo-cego, controlado por placebo, com 42 pacientes em HD, nos grupos AR (n = 22) ou placebo (n = 20). Os pacientes receberam, alternadamente, biscoitos e sachês com 16 g/dia de AR ou polvilho - 4 semanas. Coletamos amostras de sangue em jejum pré-diálise e medimos níveis séricos de AIA por cromatografia líquida de alta eficiência. Isolamos e processamos as células mononucleares do sangue periférico para avaliar expressão AhR mRNA e NF-κB por PCR quantitativo em tempo real. Avaliamos parâmetros antropométricos, bioquímicos e ingestão alimentar.

**Resultados:**

31 pacientes, 15 AR e 16 no placebo. Apesar de não apresentarem alteração significativa nos níveis de AIA, nas expressões de AhR ou NF-κB mRNA pós- suplementação com AR, foi verificada uma correlação positiva (r = 0,48; p = 0,03) entre AIA sérico e expressão de AhR na linha basal.

**Conclusão:**

Embora a suplementação com o prebiótico de AR não tenha influenciado os níveis de AIA ou a expressão de AhR, sua associação positiva reforça possível interação entre eles.

## INTRODUCTION

The aryl hydrocarbon receptor (AhR) is a transcription factor allocated in the basic helix-loop-helix subgroup of the Per-Arnt-Sim family of transcription factors, a class of proteins that are considered environmental sensors, widely expressed at central and systemic level^1^. AhR was firstly identified as a transcription factor related to the metabolism of xenobiotics, with the main activator binder being dioxins^2^. Since then, it has been extensively studied as a mediator of cellular responses against environmental toxicants. The latest findings show that in addition to dioxins, other endogenous ligands are capable of activating it, thereby associating AhR to the various physiological pathways needed to maintain homeostasis in different tissues and cell types of animals and humans^3^^-^ 7. Among those endogenous ligands are tryptophan metabolism-derived uremic toxins, such as indole-3-acetic acid (IAA)^8^.

Tryptophan, an essential amino acid, is present in animal foods such as meat, milk, and eggs and when it reaches the colon, tryptophan can be fermented by the microbiota and produce IAA^9^^,^ 10. In a healthy individual, uremic toxins are easily excreted by the kidneys, but in chronic kidney disease (CKD) their clearance is impaired, and dialysis is not able to filter them because they are strongly bounded to serum albumin^11^.

Uremic toxins have been associated with inflammation and oxidative stress, as well as progression of CKD^12^^,^ 13 and higher incidence of cardiovascular diseases (CVD)^14^^-^ 16. Among the uremic toxin, IAA has been associated with the pathogenesis of CVD by stimulating the production of procoagulant tissue factors^17^ and by the activation of pro-inflammatory mechanisms^16^^,^ 18. Indeed, IAA - a tryptophan metabolite - is picked up into the cell by transporters of organic anions and binds to AhR complex, activating it and triggering inflammatory signaling response trough AhR binding^19^. In addition, when the uremic toxins are bound to AhR, the hypoxia-induced factor 2 alpha (HIF2α) is not translocated to the nucleus and the gene erythropoietin expression is reduced leading to anemia^20^. It is important to notice that AhR activation also involves many physiological functions like fetal development and CD4+ T-cell differentiation^19^.

Uremic toxin-AhR complex translocate into the nucleus and dissociates from the stabilizer protein complex to be able to form a heterodimeric complex with aryl hydrocarbon nuclear translocator inducing the production of free radicals and inflammatory cytokines^21^^,^ 22.

Studies have suggested that supplementation of dietary fibers with prebiotic function, like resistant starch (RS), may exert a possible modulatory effect on the intestinal microbiota in CKD patients, reducing the production of uremic toxins, thereby reducing inflammation and oxidative stress^23^^-^ 25. In addition, we recently reported that RS supplementation for 4 weeks can ameliorate uremic toxins levels, oxidative stress markers, and IL-6^26^. Thus, the aim of this study was to investigate the possible effects of RS supplementation on IAA plasma levels and AhR mRNA expression in the CKD.

## SUBJECTS AND METHODS

### STUDY POPULATION

The detailed characteristics of patients has been published elsewhere^26^. Patients received a diet prescription of 1.2 to 1.4 g protein/kg/day and 30-35 kcal/kg/day, as recommended for HD patients, according to their needs. Dietary monitoring was performed by the nutritionist of the clinic and directed according to the biochemical exams of the patients. Drugs used by both groups were similar, including iron, erythropoietin, antihypertensive, and phosphorus chelators. The sample size calculation was performed using the G-Power software, with a test power of 80%, considering the CRP as the main outcome, 5% significance level (two-tailed), and 17 patients for each group.

### STUDY DESIGN

This longitudinal, randomized, double-blind, and placebo-controlled clinical trial was conducted between 2016 and 2017 in Rio de Janeiro, Brazil, and was a secondary analysis of the RS supplementation study. Blood samples from HD patients, recruited into the study previously described^26^ were analyzed for IAA plasma levels and AhR mRNA expression.

Briefly, patients were randomized in two groups, RS and placebo, to receive during 4 weeks the corresponding supplementation (16 g Hi-Maize 260^®^, Ingredion or manioc flour, Yoki). Patients received the respective supplementation in the intercalated form of cookies (1 package/day with 9 units, delivered during the dialysis session for on-site consumption) and powder (1 sachet/day for consumption on non-dialysis days). Cookies and sachets were prepared biweekly in the Laboratory of Nutrition and Dietetics of the Faculty of Nutrition - UFF, and were identical in color, appearance, size, and shape, and were packed in identical plastic packaging.

Blood collection, anthropometry, and food recall were performed at baseline and after 4 weeks of supplementation period as previously described^26^. This project design was approved by the Research Ethics Committee of the School of Medicine and the Antonio Pedro University Hospital and was assigned the permit n° CAAE 47703315.6.0000.5243. This study was also registered in the Clinical Trials service of the US National Institutes of Health (clinicaltrials.gov - NCT02706808). All procedures were in accordance with the principles of the Declaration of Helsinki.

### ANALYTIC PROCEDURES AND BIOCHEMICAL ANALYSES

Fasting blood samples were obtained prior to HD procedure. The aliquots of whole blood were divided to obtain plasma and serum and for isolation of peripheral blood mononuclear cells (PBMCs).

To obtain plasma and serum, blood was centrifuged (15 min, 3500 rpm, 4ºC), and then stored at -80ºC until further analysis. IAA plasma levels were measured by high performance liquid chromatography as previously described27. Serum levels of phosphorus, potassium, creatinine, and albumin were analyzed using Bioclin^®^ kits (Catalog #K020, K131, K016, K040 respectively) by automatic biochemical analyzer. PBMCs were isolated and analyses of real time quantitative polymerase chain reaction (rt-PCR) were performed to evaluate the AhR and NF-κB mRNA expression according Cardozo et al.28. TaqMan gene expression assays (Applied Biosystems^®^) were used for detection of AhR mRNA (Hs00169233_m1), NF-κB mRNA (Hs00765730_m1), and the control gene, GAPDH (Hs02758991_g1). PCR amplification was performed with the ABI Prism 7500 (Applied Biosystems^®^) sequencing detection system and cycle condition standards. The expression of the levels of AhR and NF-κB was normalized as a function of the GAPDH and the level of expression was calculated using the detection delta threshold cycle (ΔΔCT) method.

In addition, routine biochemical parameters, including hemoglobin, hematocrit, urea pre and post-dialysis, were measured according to standard methods of the routine clinical laboratory in the HD clinic.

### STATISTICAL ANALYSES

According to the Kolmogorov-Smirnov test, results were reported as mean ± standard deviation (SD) or median (interquartile range). Student’s t-test or Mann-Whitney test was used to evaluate differences between means; Pearson’s or Spearman’s correlation coefficients were used to evaluate correlations. The supplementation effect (∆) for each variable was defined as the within patient difference between the end and the baseline of RS or placebo supplementation. The tests were set with 95% confidence values (p <0.05) and were considered significant. Statistical analyzes were performed using the Statistical Package for Social Sciences version 23.0.

## RESULTS

Of the 43 HD patients selected for the study, 22 were allocated in the RS group and 20 in the placebo group. One patient died before randomization and 31 ended the supplementation period, 15 in the RS group (47% men, 56.0 ± 7.5 years; 50.0 ± 36.6 months of HD) and 16 in the placebo group (69%, 53.5 ± 11.5 years and 44.3 ± 26.4 months of HD). The reasons why patients were lost in this study have already been described^26^.

Clinical characteristics, and biochemical and anthropometric parameters have been previously reported; neither at baseline nor at the end of the study differences were observed. Although there was no significant change in dietary habits after the supplementation period in both groups, the group that consumed RS increased fiber intake (from 18.6 ± 7.1 to 34.4 ± 7.9, p=0.0001)^26^.

**Figure 1:**
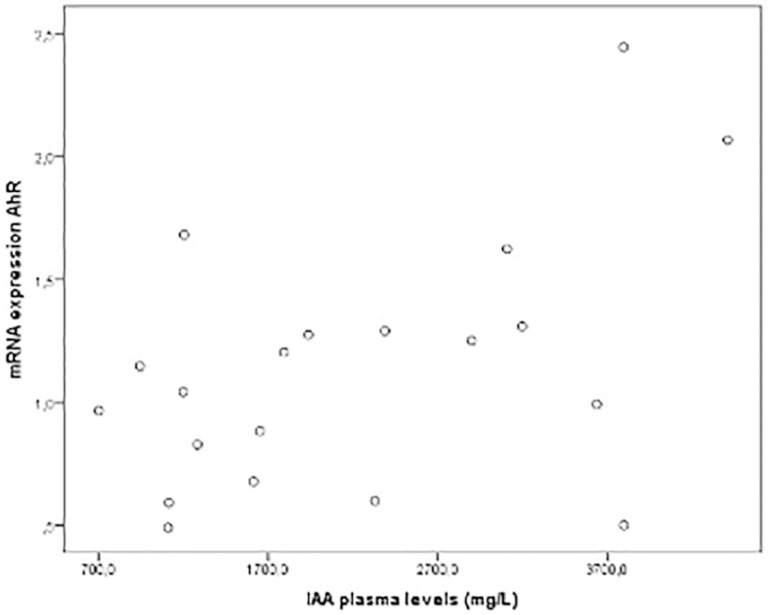
Correlation between plasma IAA levels and AhR mRNA expression in chronic kidney disease patients on hemodialysis (r=0.48; p=0.03).

Furthermore, there was no significant difference in IAA plasma levels or in AhR mRNA expression in both groups after the 4-week intervention. There was a tendency to reduction of NF-κB mRNA expression in the RS group (Table 1). Nevertheless, a positive correlation (r=0.48; p=0.03) was observed between IAA plasma levels and AhR mRNA expression at baseline (patients included in both groups) (Figure 1).

**Table 1:** Effects of resistant starch (RS) and placebo supplementation on AhR and NF-κB mRNA expression and IAA plasma values in chronic kidney disease patients on hemodialysis.

Parameters	RS group (n=15)				Placebo group (n=16)				
	*Baseline*	Post	p-value	Δ	*Baseline*	Post	p-value	Δ	p-value of Δ
AhR expression	1.1 ± 0.5	1.1 ± 0.5	0.84	0.3 (-0.4; 0.5)	1.1 ± 0.5	1.0 ± 0.5	0.29	-0.1 (-0.7; 0.3)	0.19
NF-κB expression	1.35 ± 0.81	0.97 ± 0.37	0.06	-0.2 (-0.6; 0.19)	1.0 ± 0.54	1.16 ± 0.64	0.49	0.04 (-0.61; 0.61)	0.18
IAA (mg/L)	2132.0 ± 1167.0	1917.0 ± 956.0	0.16	6.6 (-427.0; 226.0)	2004.0 ± 1035.0	1840.0 ± 908.0	0.59	-194.0 (-1254.0; 520.0)	0.89

AhR, aryl hydrocarbon receptor; NF-κB, Nuclear factor-κB; IAA, indole-3-acetic acid. Data are presented as mean ± SD or median (25-75th percentile).

## DISCUSSION

The present study revealed that 4 weeks with 16 g of RS supplementation was not able to reduce uremic toxin IAA plasma levels or AhR and NF-κB mRNA expression in HD patients. Nevertheless, a positive correlation was observed between IAA plasma levels and AhR mRNA expression, which reinforces the idea that higher concentrations of uremic toxins, specifically IAA, may be involved with activation of the AhR.

The present clinical trial was the first to evaluate the effect of a prebiotic, specifically RS, by the concentration of the IAA uremic toxin and the possible activation of the AhR receptor, which was identified as a major uremic toxin receptor, and could therefore play a central role in the activation of the inflammatory pathway in CKD^29^. In our previous review, we discussed the AhR expression and its association with uremic toxins and inflammation in CKD patients^19^.

To our knowledge, only one study examined the effects of fermentative soluble fiber supplementation on the inflammatory state of HD patients^30^, and only two studies evaluated the use of RS supplementation in HD patients^31^^,^ 26.

In the Khosroshahi study^31^, the RS supplementation was able to reduce significantly TNF-α, IL-6, and malondialdehyde plasma levels. Reinforcing these findings, our pilot study^26^ observed a reduction of IL-6 and thiobarbituric acid reactive substances (TBARS) plasma levels, as well as indoxyl sulfate plasma levels after four weeks of 16 g RS supplementation. Further, a positive correlation between IS and IL-6 was observed. Similar findings, such as reduction on uremic toxins’ levels and therefore inflammation and oxidative stress markers, have been found in previous CKD rat studies fed with RS^24^^,^ 25. In addition, in the present study there was a tendency in NF-kB expression reduction. Vaziri et al. (2014)^24^ observed reduction of nuclear factor kappa-B (NF-kB) expression after supplementation with resistant starch 2 supplementation in nephrectomized rats. The mechanisms by which the RS can modulate the NF-kB expression is unclear; however, the hypothesis that this prebiotic can modulate the gut microbiota, reducing toxins and increasing short-chain fatty acids production may be accepted.

Previous studies have already evaluated levels of uremic toxins and their role in the pathogenesis of CKD and cardiovascular events, which are a cause of death among CKD patients. Among these toxins, IS is the main precursor of cardiovascular diseases, associated with general and cardiovascular mortality^15^, and with all-cause mortality in CKD patients, particularly for those on dialysis^16^.

IAA and uremic toxins relationship have been explored in the CKD^16^^,^ 23 ^,^ 32 ^,^ 33. In a clinical trial, IAA was associated with an increase in mortality and cardiovascular events in CKD patients with IAA levels > 3.73 µM when compared to patients with lower levels (IAA <= 3.73 µM). To confirm such association, it was observed by cell culture that IAA induced oxidative stress by increasing ROS synthesis and inflammation via AhR / p38MAPK / NF- κB, increasing inflammatory cytokines, and activating ciclo-oxigenase-2^16^.

Although there was no significant reduction in IAA levels in our study, Sirich et al.^23^ reported that after the intervention with 15 g of amylose-rich corn starch (Hi-maize 260) via sachets for 6 weeks the IS free plasma level was significantly reduced when compared to the control group. It is worth mentioning that although our study had a shorter intervention time (4 weeks), the amount of RS administered per day was higher (16 g). In addition, Ramos et al. did not observed reduction of IAA after supplementation with a prebiotic (FOS) in pre-dialysis patients, agreeing with our results^33^.

In this context, reducing the concentration of uremic toxins in CKD by reestablishing the intestinal microbiota with prebiotic supplementation emerges as a non-pharmacological strategy to decrease the activation of the inflammatory cascade via activation of AhR^23^^-^ 25. The mechanisms of action of prebiotics, specifically RS, in the human intestine are complex and several hypotheses have been proposed to explain its beneficial effects on the intestinal and systemic health of HD patients. RS is fermented in the large intestine by symbiotic bacteria (Firmicutes, Bacteroidetes, and Actinobacterium), which have enzymatic activity capable of cleaving the α 1,4 and α 1,6 bonds of the RS resulting in the products of this fermentation, short chain fatty acids (SCFAs) (methane, hydrogen, carbon dioxide), organic acids (lactate, succinate, and fumarate), and alcohols (methanol and ethanol)^34^. Vaziri et al*.* 35 reported that after RS2 supplementation there was an increase in the population of symbiotic bacteria, including *Bifidobacterium* and *Faecalibacterium prausnitzii*, which have anti-inflammatory potential.

The SCFAs promotes the reduction of intestinal pH, thus modulating the intestinal microbiota and reducing uremia^36^^,^ 37. In addition, SCFAs are energetic substrates for T- regulatory lymphocytes, thereby enhancing immunity, and for the colonocytes, restoring mucosal barrier integrity^37^. Once this barrier is in full, it becomes more selective and does not allow the passage of uremic toxins, bacteria and their lipopolysaccharide cell wall components to bloodstream, thereby reducing inflammation present in CKD patients^38^. Among other effects of RS is the improvement of insulin sensibility^39^ and reduction of lipid profile^40^ and adiposity^39^.

Regarding the limitations of the study, the small sample size and the fact that some participants withdrew from the study may have prevented the emergence of other results with statistical significance. Furthermore, it was not possible to evaluate the expression of known receptor-activated genes such as CYP1A1, CYP1A2, and CYP1B1 or even perform a western blotting technique to evaluate AhR receptor and NF-kB protein.

Although these studies suggest a possible modulating effect of the intestinal microbiota in CKD patients by reducing the production of uremic toxins and with this decreasing the activation of the inflammatory cascade and lowering oxidative stress, the prebiotic action in these patients is still unclear. Indeed, this is the first evidence of the impact of prebiotic supplementation, specifically RS, on AhR expression.

## CONCLUSION

In summary, data from this study suggest that supplementation with prebiotic RS does not influence IAA plasma levels nor AhR and NF-κB mRNA expression. Furthermore, the study suggests that IAA levels are positively associated with AhR activation. Further studies are needed to explore the prebiotic supplementation on uremic toxin reduction and its relationship with the possible activation of AhR in this population.
